# Cost-effectiveness analysis of granulocyte colony-stimulating factors for the prophylaxis of chemotherapy-induced febrile neutropenia in patients with breast cancer in Taiwan

**DOI:** 10.1371/journal.pone.0303294

**Published:** 2024-06-10

**Authors:** Tzu-Hsuan Tseng, Shao-Chin Chiang, Jason C. Hsu, Yu Ko

**Affiliations:** 1 Department of Clinical Pharmacy, School of Pharmacy, College of Pharmacy, Taipei Medical University, Taipei, Taiwan; 2 Department of Pharmacy, College of Pharmaceutical Sciences, National Yang Ming Chiao Tung University (Yang Ming Campus), Taipei, Taiwan; 3 Center for Advanced Pharmacy Education, Koo Foundation Sun Yat-Sen Cancer Center, Taipei, Taiwan; 4 International PhD Program in Biotech and Healthcare Management, College of Management, Taipei Medical University, Taipei, Taiwan; 5 Research Center for Pharmacoeconomics, College of Pharmacy, Taipei Medical University, Taipei, Taiwan; Qatar University, QATAR

## Abstract

**Objectives:**

To examine the cost-effectiveness of using granulocyte colony-stimulating factor (G-CSF) for primary or secondary prophylaxis in patients with breast cancer from the perspective of Taiwan’s National Health Insurance Administration.

**Methods:**

A Markov model was constructed to simulate the events that may occur during and after a high-risk chemotherapy treatment. Various G-CSF prophylaxis strategies and medications were compared in the model. Effectiveness data were derived from the literature and an analysis of the National Health Insurance Research Database (NHIRD). Cost data were obtained from a published NHIRD study, and health utility values were also obtained from the literature. Sensitivity analyses were performed to assess the uncertainty of the cost-effectiveness results.

**Results:**

In the base-case analysis, primary prophylaxis with pegfilgrastim had an incremental cost-effectiveness ratio (ICER) of NT$269,683 per quality-adjusted life year (QALY) gained compared to primary prophylaxis with lenograstim. The ICER for primary prophylaxis with lenograstim versus no G-CSF prophylaxis was NT$61,995 per QALY gained. The results were most sensitive to variations in relative risk of febrile neutropenia (FN) for pegfilgrastim versus no G-CSF prophylaxis. Furthermore, in the probabilistic sensitivity analysis, at a willingness-to-pay threshold of one times Taiwan’s gross domestic product per capita, the probability of being cost-effective was 88.1% for primary prophylaxis with pegfilgrastim.

**Conclusions:**

Our study suggests that primary prophylaxis with either short- or long-acting G-CSF could be considered cost-effective for FN prevention in breast cancer patients receiving high-risk regimens.

## Introduction

Febrile neutropenia (FN), which refers to life-threatening low neutrophil counts accompanied by fever, is the most common side effect of chemotherapy. FN is defined as an absolute neutrophil count (ANC) of less than 500 cells/μL or a count expected to fall below 500 cells/μL within the next 48 hours accompanied by a single oral temperature of ≥38.3°C or a temperature ≥38°C for an hour [1]. The incidence rate of FN was estimated to be 34.2 cases per 1,000 chemotherapy-treated patients in the United States [2]. The FN rates vary by chemotherapy regimen and other risk factors, e.g., patient age and tumor type [3]. FN is the major cause of morbidity and mortality during chemotherapy [4, 5], and infection is one of the undesired disorders that 20%-30% of patients diagnosed with FN encounter [6]. Another FN-related concern is chemotherapy dose modifications and delayed treatment [7, 8]. For patients with potentially curable cancer, e.g., early-stage breast cancer, these unplanned modifications can compromise survival, as higher relative dose intensity (RDI) improves both disease-free survival and overall survival [9–11]. Furthermore, FN has a substantial economic impact resulted from the costs of prolonged hospital stays [12, 13]. In Taiwan, one retrospective claims database analysis was undertaken to estimate the total costs of cancer-related adverse events [14]. Their results showed that the median total healthcare costs of FN per patient were New Taiwan Dollar (NT$) 39,974.

Currently, granulocyte colony-stimulating factor (G-CSF) is widely used for FN prevention, and it can also shorten the duration of and decrease the severity of neutropenia [15, 16]. Furthermore, with G-CSF prophylaxis, subsequent FN-related infection rates and hospitalizations decrease as well, and RDI is improved [1, 17]. Primary prophylaxis and secondary prophylaxis are the two prophylactic strategies. According to the international guideline recommendations from the National Comprehensive Cancer Network (NCCN) and the European Organisation for Research and Treatment of Cancer (EORTC), primary prophylaxis is recommended when the regimen’s anticipated FN risk is high (>20%) or when its risk is intermediate (10%-20%) with at least one risk factor, e.g., age >65 years [1, 17]. However, under Taiwan’s National Health Insurance (NHI) system, primary prophylaxis is not reimbursed for patients with solid tumors. To better understand the cost-effectiveness of secondary prophylaxis with various G-CSF products and to explore whether it is cost-effective to expand reimbursement coverage to primary prophylaxis, we conducted a cost-effectiveness analysis of G-CSF used as primary and secondary prophylaxis in patients with breast cancer from the perspective of Taiwan’s National Health Insurance Administration (NHIA). All G-CSFs that are reimbursed by the NHIA, including biosimilars, were included in the analysis. Breast cancer was selected as it is one of the most common cancers, and most of the patients are in early stages and receive adjuvant chemotherapies requiring the use of G-CSFs to maintain RDI.

## Methods

### Study population and G-CSF prophylaxis strategies

The hypothetical cohort consisted of women diagnosed with early-stage breast cancer who received a high-risk chemotherapy regimen with a risk of FN greater than 20%. The chemotherapy regimen was given for 6 cycles (18 weeks), with a cycle interval of three weeks. In the base case, the age was set at 56 years, which represents the median age of women diagnosed with breast cancer in Taiwan [18].

Various G-CSF prophylaxis strategies were evaluated, including no prophylaxis, primary prophylaxis, and secondary prophylaxis. All NHI-reimbursed G-CSF reference and biosimilar drugs, including pegfilgrastim, filgrastim, and lenograstim, were considered in the model. With the primary prophylaxis strategy, a G-CSF was given in the first and subsequent cycles whereas in the secondary prophylaxis strategy, a G-CSF was given in subsequent cycles only if fever or neutropenic events occurred in a prior cycle.

### Model structure

**Fig 1:**
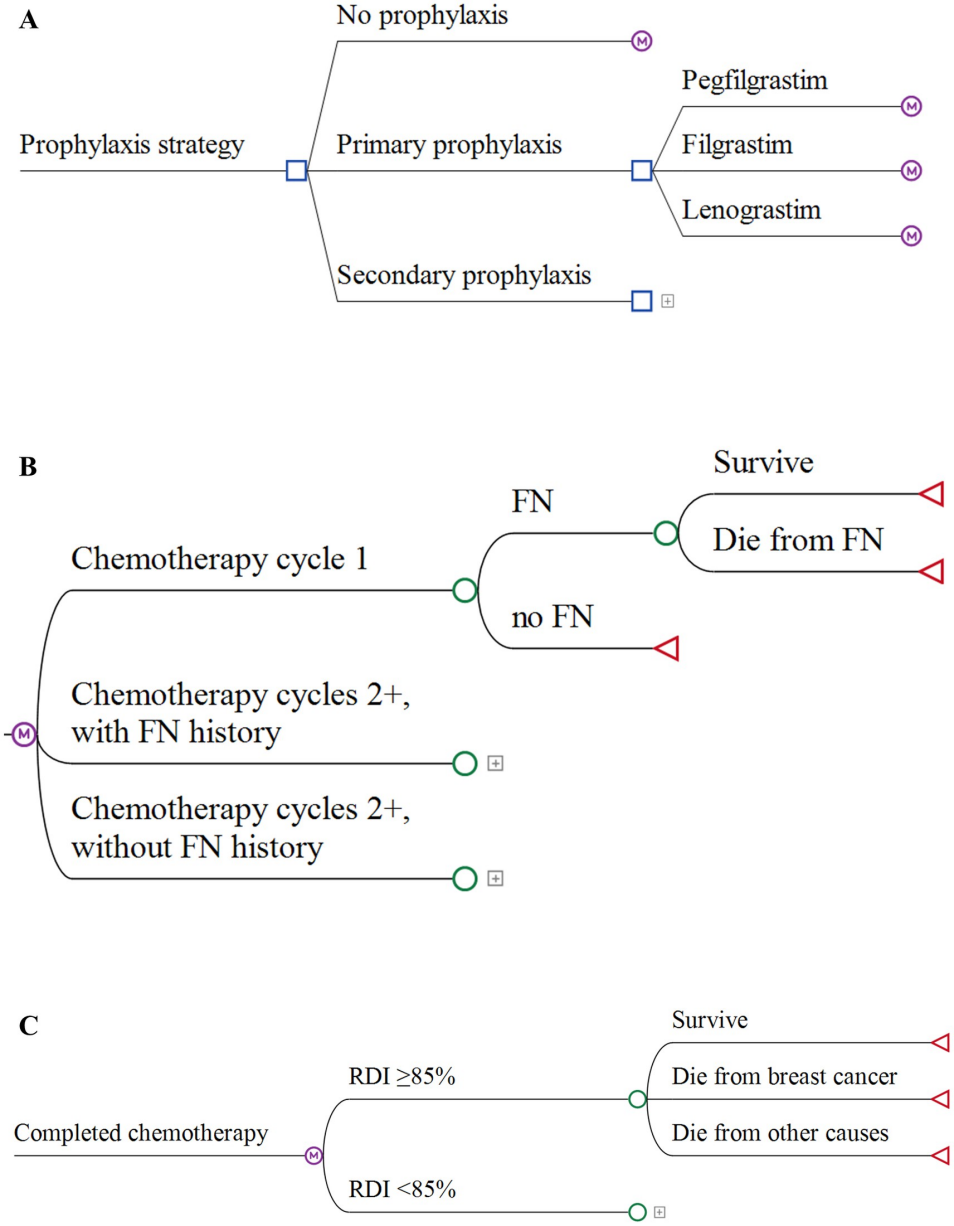
Model structure. (a) Chemotherapy period: G-CSF prophylaxis strategies; (b) Chemotherapy period: cycles 1–6 (cycle length = 3weeks); (c) Post-chemotherapy period (cycle length = 1 year).

A Markov model was constructed to examine the cost-effectiveness of G-CSF prophylaxis from the perspective of Taiwan’s NHIA. A lifetime time horizon was adopted in the analysis. In the base case, it was assumed that G-CSF reduces FN events and FN-related mortality and also improves long-term survival. Only G-CSF reference drugs were considered in the base-case analysis, and biosimilars were considered in scenario analysis. The Markov model was adapted from previous studies [19–22] and was constructed to simulate the events that may occur during high-risk chemotherapy treatment and after the completion of chemotherapy treatment, including FN events, FN-related mortality, and the impact on RDI and survival. The model consisted of two phases: (1) the chemotherapy period and (2) the post-chemotherapy period (Fig 1).

In the chemotherapy period, a prophylaxis strategy and specific G-CSF were selected at the time when the patient cohort entered the model. All patients then initiated the chemotherapy treatment. In this period, patients may or may not have experienced an FN event during each cycle, and those who experienced FN had a certain possibility of dying from FN. Afterward, patients would continue with subsequent cycles for a total of six cycles. The cycle length was set at three weeks, representing the base-case chemotherapy interval. After completing the chemotherapy course, patients were then classified based on the received RDI and were followed up until the entire cohort transitioned to the dead state. In the post-chemotherapy period, the cycle length was set at one year.

### Clinical parameters

Clinical parameters and the base-case estimates are summarized in Table 1. We used the National Health Insurance Research Database (NHIRD) to examine the effectiveness of short-acting G-CSFs in secondary prophylaxis. The study was approved by the Taipei Medical University Joint Institutional Review Board (TMU-JIRB No. N202212012). The study population was adult women (age ≥20 years) diagnosed with breast cancer who received at least four cycles of the same high-risk chemotherapy regimens from January 1, 2012, to December 31, 2019. The regimens selected in our analysis were those classified as high-risk by the NCCN and EORTC guidelines (see S1 Table). To meet the model assumptions, only patients who received chemotherapy with an interval of twenty-one days or more were included in the analysis. Patients who had G-CSF prophylaxis in the first cycle and who developed any of the following risk factors for FN one year prior to or during chemotherapy were excluded: receiving hematopoietic stem cell or bone marrow transplantation, diagnosis of human immunodeficiency virus, or receiving radiation therapy. Prophylactic use was defined as the administration of G-CSF before the diagnosis of FN and within seven days after initiation of the chemotherapy. Due to the absence of the International Classification of Diseases code for FN, a broad definition, which incorporated a diagnosis of fever, neutropenia, or infection, was adopted to identify FN events [23]. The analysis was performed based on each individual chemotherapy cycle independently. In order to avoid antibiotics’ effect of FN prevention, the cycles were excluded if antibiotics were used for FN prevention. The outcomes of interest were the incidence of FN in each cycle, and the analysis results were used to calculate the relative risk (RR) of FN for filgrastim and lenograstim compared to the no-prophylaxis group. The calculated RR values were then applied to the model. Our model assumed that the effectiveness of short-acting G-CSF in the first cycle was comparable to that in cycle 2. Therefore, the incidence of FN with G-CSF prophylaxis in the first cycle was estimated by multiplying the RR of FN in cycle 2 by the baseline risk of FN. Furthermore, the RR of FN in cycle 3 was applied to estimate the incidence of FN with G-CSF prophylaxis in subsequent cycles (i.e., cycle 3 and beyond). As the number of patients who used pegfilgrastim for prophylaxis observed in the NHIRD analysis was very small, the FN prevention data for pegfilgrastim prophylaxis were obtained from von Minckwitz et al. [24]. An RR of 0.334 for FN events in pegfilgrastim prophylaxis compared to no G-CSF prophylaxis was calculated based on their study results.

**Table 1:** Summary of model parameters.

Parameters	Base-case value	Range	Distribution	References
**FN-related parameters**				
FN baseline risk, cycle 1	0.364	0.293–0.436	Beta	NHIRD
RR of an FN event, cycles 2+ vs. cycle 1	0.46	0.4203–0.5034	Log-normal	[21]
RR of an FN event, with vs. without FN history	2.4	2.2–2.6	Log-normal	[19]
FN-related mortality	0.036	0.029–0.043	Beta	[22]
**Effectiveness of G-CSF (RR of an FN event)**				
Filgrastim vs. no G-CSF use, cycles 1–2	0.640	0.5077–0.8076	Log-normal	NHIRD
Filgrastim vs. no G-CSF use, cycles 3+	0.575	0.4477–0.7379	Log-normal	NHIRD
Lenograstim vs. no G-CSF use, cycles 1–2	0.452	0.2767–0.7393	Log-normal	NHIRD
Lenograstim vs. no G-CSF use, cycles 3+	0.609	0.3944–0.9398	Log-normal	NHIRD
Pegfilgrastim vs. no G-CSF use	0.334	0.2097–0.5319	Log-normal	[20]
**Relative dose intensity**				
Probability of RDI<85%, age <65 years and without FN history	0.247	0.149–0.345	Beta	[18, 25]
OR of RDI <85%, with vs. without FN history	1.58	1.20–2.10	Log-normal	[25]
HR for long-term mortality, RDI<85% vs. RDI ≥85%	1.73	1.17–2.55	Log-normal	[26]
**Health utility values**				
Receiving chemotherapy	0.78	0.728–0.832	Beta	[27]
FN hospitalization (decrement)	0.36	0.289–0.431	Beta	[28]
Surviving breast cancer: years 1–5	0.84	0.791–0.889	Beta	[30]
Surviving breast cancer: years 5+	0.91	0.893–0.927	Beta	[29]
**Costs**				
Pegfilgrastim 6mg, per injection (NTD)	16,626	13,301–16,626	-	NHIA [32]
Pegfilgrastim biosimilar 6mg, per injection (NTD)	14,457	-	-	NHIA [32]
Filgrastim 300 mcg, per injection (NTD)	1673	1338–1673	-	NHIA [32]
Filgrastim biosimilar 300 mcg, per injection (NTD)	1559	-	-	NHIA [32]
Lenograstim 250 mcg, per injection (NTD)	1674	1339–1674	-	NHIA [32]
Costs of FN, per event (NTD)	8287	6630–9944	Gamma	[33]
Days of short-acting G-CSF use	4	-	-	NHIRD
Length of stay for FN (days)	10.53	7.39–13.67	Gamma	[34]

FN indicates febrile neutropenia; G-CSF, granulocyte colony-stimulating factor; HR, hazard ratio; NHIA, National Health Insurance Administration; NHIRD, National Health Insurance Research Database; NTD, New Taiwan Dollars; OR, odds ratio; RDI, relative dose intensity; RR, relative risk.

The baseline FN risk in the first cycle was also derived from our NHIRD analysis. The observed FN incidence in the first cycle among the no-prophylaxis group was 36.4%. The probability of an FN event varies across cycles, with the highest incidence observed in the first cycle. Patients who have experienced an FN event in a prior cycle are more likely to experience another FN event compared to those without FN history. In the subsequent cycles (i.e., cycles 2 to 6), the probability of FN was calculated by multiplying the FN baseline risk by the RR of FN in subsequent cycles. If patients had experienced FN in a prior cycle, the RR of FN with an FN history was incorporated in the calculation of FN risk. Specifically, an RR of 0.46 was applied to subsequent cycles for FN risk calculation [25] whereas an RR of 2.4 was applied to patients with an FN history [23].

### Mortality

The FN-related mortality was assumed to be 3.6%, which data was obtained from a previous study investigating the mortality rate in breast cancer patients hospitalized for FN [26]. In addition, an estimated average annual mortality rate of 2.02% for patients with breast cancer was derived from the 10-year overall survival rate of breast cancer reported by the Health Promotion Administration [27]. We assumed that the mortality rate of breast cancer patients after 10 years was the same as that of the general population of the same age and gender, and the age-and gender-specific mortality rate data was obtained from the Ministry of the Interior [28].

### Relative dose intensity

After the completion of the chemotherapy treatment, patients were categorized into two groups based on their received RDI: RDI ≥85% or RDI <85%. Patients who are over 65 years old or have a history of FN are less likely to achieve optimal RDI (i.e., RDI ≥85%) [29]. The odds ratio (OR) for an RDI <85% in patients with an FN history compared to those without was obtained from Shayne et al. [29]. The reported OR of 1.58 for reduced RDI (i.e., RDI <85%) was then converted to RR. If the patient had experienced FN events during chemotherapy, the adjusted probability of RDI <85% was applied, which value was calculated by multiplying the RR for reduced RDI by the baseline probability of RDI <85%. A hazard ratio (HR) of 1.73 for mortality as a result of reduced RDI was applied to calculating the mortality of patients receiving RDI <85% in the model [30].

### Health utilities

The utilities associated with receiving chemotherapy, FN hospitalization, and surviving from breast cancer were incorporated into the model (Table 1). Most utility values were obtained from previous studies conducted in Taiwan [31–33], except for the utility for breast cancer survivors in the first five years, which was derived from an earlier Asian study [34]. The utility values were measured by using the time trade-off technique, the standard gamble technique, or the five‐level EuroQol‐5 dimensions questionnaire. These utilities were multiplied by the chemotherapy cycle interval of three weeks and the length of stay for FN to calculate the quality-adjusted life years (QALYs) during chemotherapy and FN hospitalization, respectively.

### Costs and resource utilization

Cost parameters and their base-case estimates are summarized in Table 1. Since our study adopted the NHI perspective, only direct medical costs were included. All costs are presented in New Taiwan Dollars (NTD) and inflated to 2022 NTD using the Consumer Price Index of healthcare [35]. Only the costs incurred during chemotherapy were considered in our study since the use of a G-CSF only affected the costs during chemotherapy, including G-CSF medication costs and costs related to FN hospitalization. The drug costs of G-CSFs were based on the most recent NHIA reimbursement prices [36]. In the model, we assumed the length of prophylactic use of short-acting a G-CSF was four days based on our NHIRD analysis. The costs related to FN hospitalization were derived from a previous retrospective claims database analysis, which estimated the costs of common adverse events (e.g., neutropenia) in breast cancer patients in Taiwan [14]. To fit the cycle length (21 days), the costs of an FN event were calculated by multiplying the estimated costs of NT$11,073 by 0.7 and then adjusted to 2022 NTD (NT$8,287). In addition, the average length of stay in cancer patients undergoing chemotherapy who were hospitalized due to hematologic adverse events, e.g., neutropenia, was estimated to be 10.53 days [37].

### Base-case analysis

In the base-case analysis, total costs were estimated for each prophylaxis strategy, and the health outcomes were measured in QALYs. A discount rate of 3% per annum was applied to health outcomes. Since all costs were incurred within the first year, no discounting was applied to the costs. The World Health Organization (WHO) guide recommends a threshold of one to three times the gross domestic product (GDP) per capita as the WTP threshold [38]. However, some researchers have raised concerns about the appropriateness of the WHO-recommended threshold [39–41]. In a recent systematic review, a range of 0.5–1.5 times GDP per capita was suggested a more reasonable WTP threshold [39]. Therefore, a threshold of one times the 2022 Taiwan’s GDP per capita, or NT$976,914, [42] was used to determine cost-effectiveness.

### Sensitivity analysis

Two scenario analyses were performed. As the extent to which G-CSF impacts overall survival remains unclear, in scenario 1, we assumed that G-CSF has no impact on long-term survival (i.e., only the reductions in FN incidence and related mortality were considered) to deal with the uncertainty. In scenario 2, we added biosimilars of filgrastim and pegfilgrastim to account for their impact on the cost-effectiveness results. The biosimilar version of lenograstim was not available in Taiwan, so it was not included in the analysis.

In addition, we performed one-way sensitivity analyses to assess the impact of individual model parameters on the results and to identify the most influential parameters. The results are presented in the tornado diagrams. For most parameters, the ranges were determined based on 95% CIs, standard errors, or plausible values derived from previous literature (Table 1). A conservative range of ±20% was used for the costs of FN and a range between -20% and 0% was used for the costs of G-CSFs. The range was not extended to +20% for the costs of G-CSFs because drug prices are continually adjusted downwards under the reimbursement policy. Moreover, probabilistic sensitivity analyses were conducted to account for overall parameter uncertainty. In the analysis, 10,000 iterations of second-order Monte Carlo simulation were performed. Cost-effectiveness acceptability curves were used to present the analysis results with the probabilities of each strategy being cost-effective illustrated at different WTP thresholds.

## Results

### Base-case analysis

In the base-case analysis, total costs, incremental costs, total QALYs, incremental QALYs, and ICERs were estimated for each strategy from the perspective of the NHIA. The results of the cost-effectiveness analysis for each strategy are presented in Table 2. No G-CSF prophylaxis had the lowest costs (NT$14,702) and the fewest benefits (13.24 QALYs) whereas primary prophylaxis with pegfilgrastim yielded the highest costs (NT$102,952) and the most benefits (13.96 QALYs). Secondary prophylaxis with lenograstim, primary prophylaxis with filgrastim, and secondary prophylaxis with pegfilgrastim were dominated by secondary prophylaxis with filgrastim or primary prophylaxis with lenograstim due to higher costs and fewer benefits. Moreover, primary prophylaxis with filgrastim was eliminated via extended dominance.

**Table 2:** Results of base-case analysis.

Strategy	Costs (NTD)	Incremental costs	QALYs	Incremental QALYs	ICER (NTD/QALY)
**No G-CSF prophylaxis**	14,702	-	13.24	-	-
**SP with filgrastim**	28,503	13,801	13.45	0.22	Extended dominance
**SP with lenograstim**	28,567	64	13.45	-0.002	Dominated
**PP with lenograstim**	46,659	31,956	13.75	0.52	61,995
**PP with filgrastim**	47,476	818	13.68	-0.07	Dominated
**SP with pegfilgrastim**	52,328	5669	13.58	-0.17	Dominated
**PP with pegfilgrastim**	102,952	56,294	13.96	0.21	269,683

G-CSF indicates granulocyte colony-stimulating factor; ICER, incremental cost-effectiveness ratio; NTD, New Taiwan Dollars; PP, primary prophylaxis; QALY, quality-adjusted life year; SP, secondary prophylaxis.

After eliminating the dominated strategies, the ICERs for three undominated strategies were calculated. Compared to no G-CSF prophylaxis, primary prophylaxis with lenograstim had an ICER value of NT$61,995 per QALY gained. Additionally, compared to primary prophylaxis with lenograstim, the ICER value of primary prophylaxis with pegfilgrastim was NT$269,683 per QALY gained. All undominated strategies were considered cost-effective options under the WTP threshold of one times Taiwan’s GDP (NT$976,914).

### Scenario analysis

The results of scenario analyses are presented in Table 3. In scenario 1, the results were similar to those of the base-case analysis, with a slight increase in the ICER values of undominated strategies. Also in scenario 1, secondary prophylaxis with filgrastim was no longer eliminated by extended dominance and had an ICER value of NT$61,613 per QALY gained compared to no G-CSF prophylaxis. In scenario 2, where biosimilars were included in the model, the ICER for using a filgrastim biosimilar as secondary prophylaxis was NT$58,494 per QALY gained. Primary prophylaxis with pegfilgrastim was dominated by its biosimilar, where the ICER was NT$207,820 per QALY gained compared to primary prophylaxis with lenograstim.

**Table 3:** Results of scenario analyses.

Strategy	Costs (NTD)	Incremental costs	QALYs	Incremental QALYs	ICER (cost/QALY)
**Scenario 1: G-CSF has no impact on long-term survival**					
**No G-CSF prophylaxis**	14,702	-	13.70	-	-
**SP with filgrastim**	28,503	13,801	13.93	0.22	61,613
**SP with lenograstim**	28,567	64	13.93	-0.002	Dominated
**PP with lenograstim**	46,659	18,155	14.20	0.27	66,596
**PP with filgrastim**	47,476	818	14.14	-0.06	Dominated
**SP with pegfilgrastim**	52,328	5669	14.06	-0.14	Dominated
**PP with pegfilgrastim**	102,952	56,294	14.39	0.19	292,562
**Scenario 2: calculations including biosimilars**					
**No G-CSF prophylaxis**	14,702	-	13.24	-	-
**SP with filgrastim biosimilar**	27,325	12,622	13.45	0.22	58,494
**SP with filgrastim**	28,503	1179	13.45	0	Dominated
**SP with lenograstim**	28,567	1242	13.45	-0.002	Dominated
**PP with filgrastim biosimilar**	44,782	17,458	13.68	0.23	Extended dominance
**PP with lenograstim**	46,659	19,334	13.75	0.30	64,516
**SP with pegfilgrastim biosimilar**	46,685	26	13.58	-0.17	Dominated
**PP with filgrastim**	47,476	818	13.68	-0.07	Dominated
**SP with pegfilgrastim**	52,328	5669	13.58	-0.17	Dominated
**PP with pegfilgrastim biosimilar**	90,039	43,381	13.96	0.21	207,820
**PP with pegfilgrastim**	102,952	12,913	13.96	0	Dominated

G-CSF indicates granulocyte colony-stimulating factor; ICER, incremental cost-effectiveness ratio; NTD, New Taiwan Dollars; PP, primary prophylaxis; QALY, quality-adjusted life year; SP, secondary prophylaxis.

### One-way sensitivity analysis

**Fig 2:**
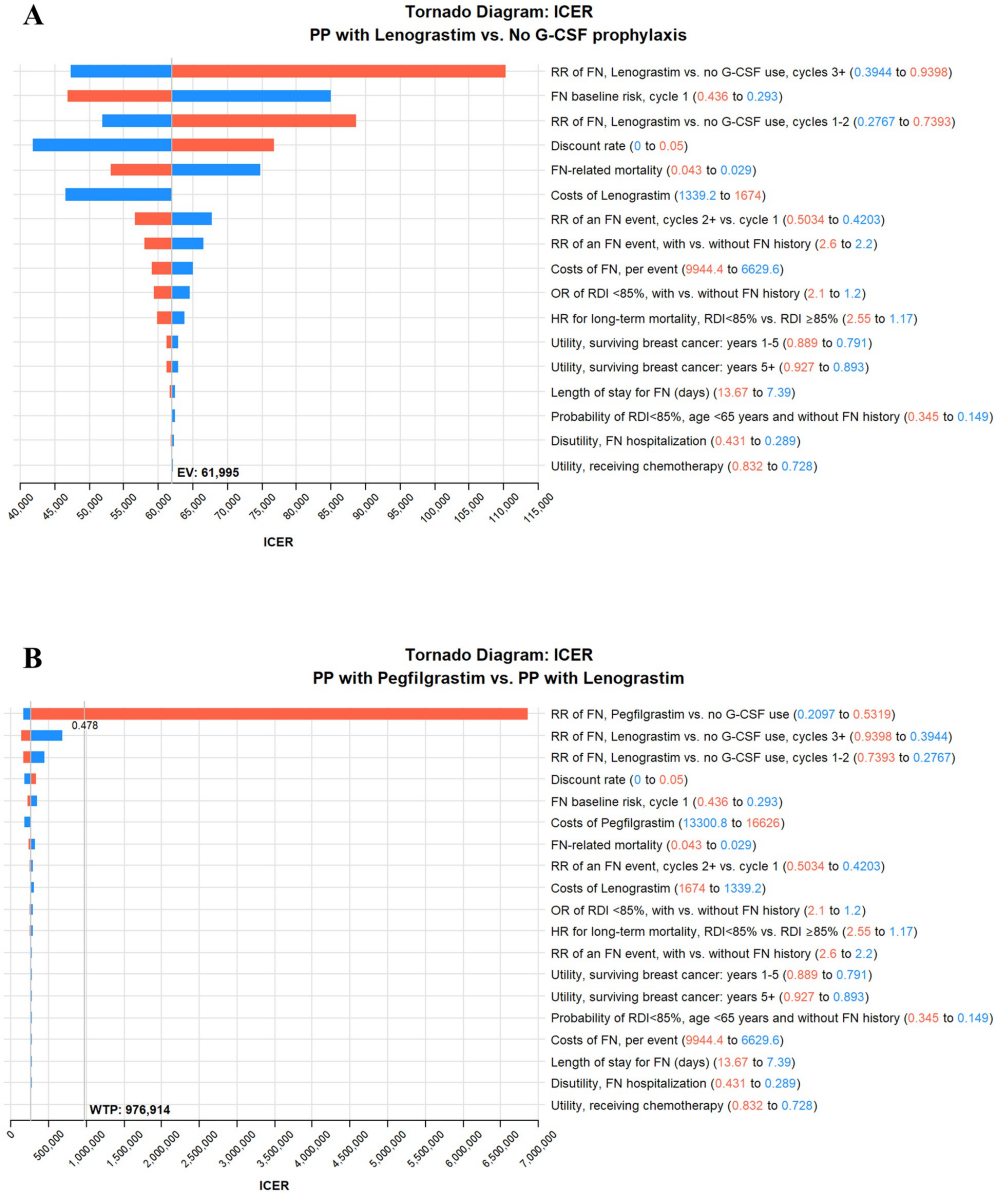
Tornado diagram of one-way sensitivity analysis. (a) PP with lenograstim compared to no G-CSF prophylaxis; (b) PP with pegfilgrastim compared to PP with lenograstim. FN, febrile neutropenia; G-CSF, granulocyte colony-stimulating factor; HR, hazard ratio; ICER, incremental cost-effectiveness ratio; OR, odds ratio; PP, primary prophylaxis; RDI, relative dose intensity; RR, relative risk.

The results of one-way sensitivity analyses are illustrated as tornado diagrams in Fig 2. In the comparison between primary prophylaxis with lenograstim and no G-CSF prophylaxis, the ICER values were most sensitive to the variations in the following parameters: RR of FN (lenograstim vs. no G-CSF prophylaxis), FN baseline risk in cycle 1, discount rate, and FN-related mortality. Nevertheless, the ICER values in all situations remained below the threshold of one times Taiwan’s GDP per capita. In the comparison between primary prophylaxis with pegfilgrastim and primary prophylaxis with lenograstim, the ICER value was most sensitive to the variations in RR of FN for pegfilgrastim versus no G-CSF prophylaxis. When the RR value exceeded 0.478, the ICER value of primary prophylaxis with pegfilgrastim would become greater than NT$976,914 per QALY gained. Variations in the other key parameters did not significantly affect the cost-effectiveness results.

### Probabilistic sensitivity analysis

**Fig 3:**
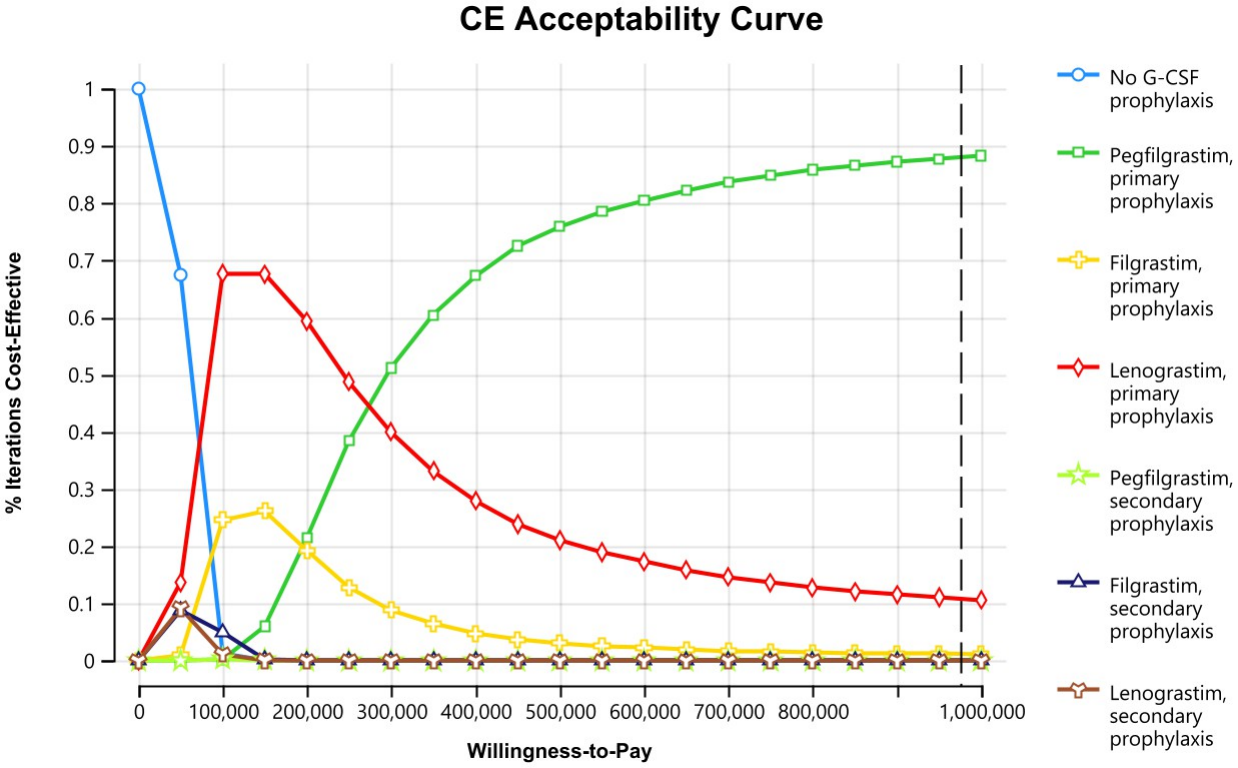
Cost-effectiveness acceptability curves. CE, cost-effectiveness; G-CSF, granulocyte colony-stimulating factor. Note: the black dashed vertical line represents one times Taiwan’s GDP per capita (NT$976,914).

The cost-effectiveness acceptability curves are presented in Fig 3. At the lower range of the WTP threshold, no G-CSF prophylaxis had the highest probability of being a cost-effective option. With a WTP threshold of between NT$100,000 and NT$250,000, primary prophylaxis with lenograstim was considered the optimal option. When the WTP threshold exceeded NT$300,000, primary prophylaxis with pegfilgrastim began to have the highest probability of being cost-effective. At a WTP threshold of one times Taiwan’s GDP per capita, the probability of being cost-effective was 88.1% for primary prophylaxis with pegfilgrastim and 10.9% for primary prophylaxis with lenograstim. The likelihood of secondary prophylaxis with any type of G-CSFs being cost-effective was low, regardless of the variation in WTP thresholds.

## Discussion

To the best of our knowledge, this is the first study incorporating long-acting G-CSF and biosimilars to evaluate the cost-effectiveness of G-CSF prophylaxis for FN in Taiwan. The effectiveness data of short-acting G-CSFs and estimated costs applied in the model were derived from real-world data in Taiwan. The results indicated that primary prophylaxis with pegfilgrastim and lenograstim are cost-effective in breast cancer patients receiving high-risk regimens.

Our findings showed that secondary prophylaxis with any type of G-CSF was dominated or extendedly dominated in the base-case analysis. There are two possible explanations for the findings. First, the reason why secondary prophylaxis with filgrastim was dominated could be due to the better prophylactic effect of lenograstim compared to filgrastim in the first two cycles of the model. In a further analysis, when the same effectiveness of filgrastim was applied to lenograstim, secondary prophylaxis with filgrastim would not be dominated and would become a cost-effective option. Second, a relatively high value of baseline FN risk was applied to our model based on the observation from the NHIRD analysis. When the risk of FN is higher, primary prophylaxis is more likely to be cost-effective or may even dominate secondary prophylaxis. Although secondary prophylaxis with filgrastim was cost-effective in the scenario analyses, the results in probability sensitivity analysis demonstrated that secondary prophylaxis was less likely to be a cost-effective option under any WTP threshold.

In our study, primary prophylaxis with filgrastim was dominated by lenograstim. The main reason is that the drug prices of the short-acting G-CSFs are quite similar in Taiwan; in addition, a more effective prophylactic benefit of lenograstim was assumed in the model based on the NHIRD analysis. Nevertheless, previous clinical trials have demonstrated that the efficacy of lenograstim in reducing the incidence of FN is similar to that of filgrastim [43]. In reality, there is generally only one short-acting drug available in a hospital. Therefore, it is suggested that primary prophylaxis with filgrastim and lenograstim should both be considered to be cost-effective options.

Our findings that primary prophylaxis is cost-effective in patients with breast cancer are consistent with previous studies [19–21, 44, 45]. For example, a previous study conducted in Taiwan evaluated the cost-effectiveness of short-acting G-CSFs in breast cancer patients and found that primary prophylaxis was cost-effective and secondary prophylaxis was a dominated strategy [45]. While the previous study only evaluated the cost-effectiveness of short-acting G-CSFs, our study further incorporated long-acting G-CSF and biosimilars into the evaluation. In addition, while the previous study was limited to a time horizon during chemotherapy, with ICERs presented as the incremental cost per FN rate avoided, our study further extended the evaluation to a lifetime horizon and took into account improvement in quality of life. Both studies reported consistent findings that primary prophylaxis was cost-effective from the NHIA perspective.

Several other studies that evaluated the cost-effectiveness of long- and short-acting G-CSFs also reported that primary prophylaxis with pegfilgrastim was cost-effective compared to 6-day filgrastim [20, 21, 44]. In addition, in the cost-effectiveness analysis conducted by Fust et al., not only were both long- and short-acting G-CSFs used as primary and secondary prophylaxis but two different durations (6 days and 11-days) of short-acting G-CSF use were also included in the comparisons [19]. Their results indicated that, compared to other prophylaxis strategies, primary prophylaxis with pegfilgrastim appears to be cost-effective.

However, conflicting findings were also reported in two previous studies that suggest secondary prophylaxis was cost-effective at the country-specific WTP threshold, whereas primary prophylaxis was likely to be cost-effective at a higher WTP threshold [22, 46]. An explanation for the difference in findings could be that a higher incidence of FN risk was applied to our study; as a result, the benefits of primary prophylaxis would be more significant. In addition, primary prophylaxis with lenograstim was considered cost-effective in our study while previous studies demonstrated that primary prophylaxis with a short-acting G-CSF was dominated by using a long-acting G-CSF [19, 22]. The inconsistent findings may have resulted from the differences in the prices of individual drugs and the differences in drug prices between short- and long-acting G-CSFs across countries, as well as the different data sources and estimates of FN prevention rates.

Our study included all available G-CSFs, including biosimilars, to provide a comprehensive evaluation of the cost-effectiveness of G-CSF prophylaxis under the healthcare system in Taiwan. Furthermore, the NHIRD, which covers over 99% of the entire population, was used to obtain effectiveness data for G-CSF prophylaxis, and the data obtained were applied to the model to better reflect the actual clinical practice and outcomes in Taiwan. Currently, the reimbursement coverage for solid tumors is restricted to secondary prophylaxis regardless of the FN risk of the given regimen. In addition, the coverage of secondary prophylaxis with pegfilgrastim is further restricted to only patients with bone marrow invasion. Our study demonstrated that primary prophylaxis with either long- or short-acting G-CSFs could be cost-effective. As for whether a long-acting or short-acting G-CSF is more favorable, several factors need to be cautiously considered, including the actual WTP threshold adopted by the NHIA, the effectiveness differential between long- and short-acting G-CSFs, the convenience of pegfilgrastim’s once-per-cycle administration, and patient preference and adherence to medication. The findings of the present study could serve as a reference for policy making in future reassessment of reimbursement coverage of G-CSFs and also for physicians’ prescribing practices.

### Limitations

There are several limitations to our study that should be noted. First, our study took the healthcare payer’s perspective; therefore, indirect costs incurred by FN such as productivity loss and informal caregiver costs were not captured in our analysis. Second, the effectiveness data for pegfilgrastim were derived from the literature, as there were few pegfilgrastim users in the NHIRD due to reimbursement restrictions. Third, due to the absence of local data, the utility of breast cancer survival within five years was obtained from a study conducted in Singapore [34]. Nevertheless, the sensitivity analysis showed that the uncertainty in utility had minimal impact on the results. Lastly, in our NHIRD analysis, patients who had received radiation therapy were excluded and the intervals of chemotherapy were limited to a minimum of three weeks. This may limit the generalizability of the results in real-world setting.

## Conclusions

In conclusion, our study suggests that primary prophylaxis with either a short- or long-acting G-CSF could be considered cost-effective in patients with breast cancer receiving high-risk regimens at the WTP threshold of one times Taiwan’s GDP per capita from the perspective of Taiwan’s NHIA. The likelihood of secondary prophylaxis being a cost-effective option is relatively low regardless of the WTP threshold used. The findings may provide valuable insights for physicians’ prescribing practices and also for future assessment of reimbursement policy, including the coverage of primary prophylaxis and the use of biosimilars.

## Supporting information

S1 Table The list of high-risk chemotherapy regimens included in the NHIRD analysis. (DOCX)
