# Supplementary material for: Cost-effectiveness analysis of granulocyte colony-stimulating factors for the prophylaxis of chemotherapy-induced febrile neutropenia in patients with breast cancer in Taiwan
Source: PLoS One. 2024 Jun 10;19(6):e0303294. doi: 10.1371/journal.pone.0303294 (PMC11164394; doi:10.1371/journal.pone.0303294)
Supplement: S1 Table — (DOCX) [file pone.0303294.s001.docx]

S1 Table. The list of high-risk chemotherapy regimens included in the NHIRD analysis

| **The list of high-risk (anticipated FN risk >20%) chemotherapy regimens** | |
| --- | --- |
| TAC (Docetaxel^1^, Doxorubicin^2^, Cyclophosphamide) | Doxorubicin^2^ + Docetaxel^1^ |
| TC (Docetaxel^1^, Cyclophosphamide) | Doxorubicin^2^ + Paclitaxel^1^ |
| TCH (Docetaxel^1^, Carboplatin, Trastuzumab) |  |

FN indicates febrile neutropenia.

^1^Docetaxel and paclitaxel are considered interchangeable in these regimen.

^2^Doxorubicin and epirubicin are considered interchangeable in these regimen.
